# Gut and Reproductive Tract Microbiota Adaptation during Pregnancy: New Insights for Pregnancy-Related Complications and Therapy

**DOI:** 10.3390/microorganisms9030473

**Published:** 2021-02-25

**Authors:** Martina De Siena, Lucrezia Laterza, Maria Valeria Matteo, Irene Mignini, Tommaso Schepis, Gianenrico Rizzatti, Gianluca Ianiro, Emanuele Rinninella, Marco Cintoni, Antonio Gasbarrini

**Affiliations:** 1 Unità Operativa Complessa (UOC) di Medicina Interna e Gastroenterologia, Dipartimento di Scienze Mediche e Chirurgiche, Fondazione Policlinico Universitario A. Gemelli IRCCS, 00168 Rome, Italy; martinadesiena@gmail.com (M.D.S.); mariavaleria31191@gmail.com (M.V.M.); irene.mignini@gmail.com (I.M.); tommaso.schepis@gmail.com (T.S.); gianenrico.rizzatti@gmail.com (G.R.); gianluca.ianiro@hotmail.it (G.I.); antonio.gasbarrini@unicatt.it (A.G.); 2 Unità Operativa Complessa (UOC) di Nutrizione Clinica, Dipartimento di Medicina e Chirurgia Traslazionale, Università Cattolica del Sacro Cuore, 00168 Rome, Italy; emanuele.rinninella@unicatt.it; 3 Scuola di Specializzazione in Scienza Dell’Alimentazione, Università di Roma Tor Vergata, 00133 Rome, Italy; marco.cintoni@gmail.com

**Keywords:** microbiota, pregnancy, vaginal microbiota, endometrial microbiota, placental microbiota, preterm birth, gestational diabetes mellitus, pre-eclampsia

## Abstract

Pregnancy is characterized by maternal adaptations that are necessary to create a welcoming and hospitable environment for the fetus. Studies have highlighted how the microbiota modulates several networks in humans through complex molecular interactions and how dysbiosis (defined as quantitative and qualitative alterations of the microbiota communities) is related to human pathologies including gynecological diseases. This review analyzed how maternal uterine, vaginal, and gut microbiomes could impact on fetus health during the gestational period. We evaluated the role of a dysbiotic microbiota in preterm birth, chorioamnionitis, gestational diabetes mellitus and pre-eclampsia. For many years it has been hypothesized that newborns were sterile organisms but in the past few years this paradigm has been questioned through the demonstration of the presence of microbes in the placenta and meconium. In the future, we should go deeper into the concept of in utero colonization to better understand the role of microbiota through the phases of pregnancy. Numerous studies in the literature have already showed interesting results regarding the role of microbiota in pregnancy. This evidence gives us the hope that microbiota modulation could be a novel strategy to reduce the morbidity and mortality related to pregnancy complications in the future.

## 1. Introduction

During pregnancy, several adaptations occur in the female organism. In fact, from fertilization until delivery, the maternal body changes and activates a series of physiological transformations to welcome the new life [1]. Several adjustments in the hemodynamic state [2] and in respiratory, cardiac [3,4], urogenital [5,6] and gastrointestinal systems [7,8] occur. The microbiota as a component of human bodies is subject to these modifications and at the same time it contributes, through the production of active metabolites, to them. The composition of microbiota is influenced by factors such as the genotype, sex, age, the immune status, and various environmental factors. Several niches of our body are colonized by microbes, but the main microbial density could be found on body surfaces that interact with the external environment such as the respiratory, urogenital, and gastrointestinal systems and the skin. The microbiome is the whole genetic heritage of the microbiota and it accounts for a total of about 3.3 million genes, able to produce millions of active metabolites that interact with complex molecular cascades in the host. The most studied microbiota belongs to the gut and it is well known that gut microbiota play multiple functions [9], including defense from external pathogens [10] and bidirectional interactions with the endocrine, metabolic [11], nervous [12,13] and immune systems [14,15]. The gut microbiota is composed of about 10^14^ microorganisms including bacteria, eukaryotes, viruses, and archaea. There are two major phyla named Firmicutes and Bacteroidetes [16] that account for 80–90% of the intestinal bacterial microbiome (the bacteriome), but there are also other numerically less represented phyla such as Proteobacteria, Verrucomicrobia, Actinobacteria and Fusobacteria. The gut microbiome has considerable interindividual and intraindividual variability depending on the surrounding environment; however, this is classified into three major subtypes according to the most represented bacterial clusters, the enterotypes [17]. The prevalence of one enterotype over the others depends on individual genes, external environment and eating habits. Particularly, Enterotype 1 is mainly composed of *Bacteroides*, Enterotype 2 by *Prevotella*, and Enterotype 3 by *Ruminococcus*. The enterotypes absolve functions that are necessary for the maintenance of intestinal eubiosis. Thanks to its variability, the microbiome of every human being is extremely unique. We should imagine the microbiota as a dynamic entity able to actively interact with the different molecular and cellular networks of our organism rather than a compartmentalized community confined in separated body niches. Eubiosis is the condition characterized by a quantitative and qualitative balance of all the microbiota components [18] and occurs when microbes positively interact with each other and with the host for the maintenance of body homeostasis. Over the entire life, from birth to the elderly, microbiota changes in response to external stressor events and to new physiological statuses, i.e., pregnancy or senescence, to guarantee the maintenance of eubiosis [19,20], showing characteristics of resistance and resilience. Resistance is the property of gut microbiota to remain stable after a disturbance from the environment; resilience, instead, defines how quickly microbiota will recover its initial functional or taxonomic composition after a perturbation. In fact, during life, the microbiota continuously adapts and dynamically responds to external stressor events to ensure homeostasis. Dysbiosis is a qualitative and/or quantitative alteration of the microbial communities with consequent impairment of all the related functions. Dysbiosis has been linked to several pathologies such as asthma [21], inflammatory bowel diseases [22,23], obesity [24,25], diabetes mellitus [26], and neurodegenerative and psychiatric disorders [12,13,27]. However, in many cases it is not clear if microbiota unbalance represents the etiology or the consequence of human pathologies and, similarly, the causal mechanism that links dysbiosis to human diseases has to be clarified. Beyond gut microbiota, the female genital tract microbiota has been largely studied in recent years. This review will focus on gut and reproductive tract microbiota variations during physiologic pregnancy and in case of pregnancy complications, particularly gestational diabetes mellitus (GDM), pre-eclampsia (PE), and preterm birth (PTB).

## 2. Materials and Methods

The literature search was performed on PubMed from inception until 26th August 2020 by two authors (M.D.S. and L.L.) independently. The following combinations of terms were searched: (microbiota OR microbiome) AND pregnancy; gut microbiota AND pregnancy; vaginal microbiota AND pregnancy; preterm birth AND dysbiosis; preterm birth AND vaginal microbiota; pregnancy complication AND dysbiosis; pre-eclampsia AND microbiota; pre-eclampsia AND dysbiosis; gestational diabetes mellitus AND microbiota; gestational diabetes mellitus AND dysbiosis. Retrieved papers were firstly selected based on title and abstracts. Only studies in humans were selected. Then, selected papers were evaluated in full text. References of pivotal reviews were manually searched to identify any missed relevant references.

## 3. Results

### 3.1. Physiologic Transformations during Pregnancy

#### 3.1.1. Gut Microbiota and Immunologic Adaptations during Pregnancy

During pregnancy, major changes have been seen in mothers’ gut microbiota. Between the first (T1) and third trimester (T3) of pregnancy [28], to support the fetus growth, there is a shift towards communities of microbes implicated in energy production and storage. In fact, there is an increase in *Akkermansia* and *Bifidobacterium* at the genus level and Firmicutes, Proteobacteria and Actinobacteria at the phylum level. *Akkermansia*, *Bifidobacterium* and Firmicutes have been associated with increased storage of energy, whereas Proteobacteria and Actinobacteria protect mother and fetus from external infections, acting as proinflammatory bacteria. Throughout the gestational period, the condition of the maternal immune system needs to be considered unique. Thanks to multiple local and systemic adaptations, the maternal body has become able to establish protection from the external environment and tolerance towards the fetus at the same time [29]. Thus, the correct term to identify the maternal immune system transformation is not “suppression” but “modulation” [30]. To better understand this concept, we should consider the fetus as a semiallograft tissue that induces immune modifications that are not characterized by weakening or depotentiation. In fact, during the gestational period, the mother must protect herself and the fetus from infections and external environment through an active and ready immune response [31]. Furthermore, some studies revealed that depletion of immune system cells determines early pregnancy termination interfering with development, implantation, and decidual formation [32,33]. During normal pregnancy, decidua contains macrophages and natural killer (NK) and regulatory T cells [34,35] and the cooperation between trophoblast and immune system cells promotes decidua invasion, oxygen and nutrient transport, angiogenesis and protection against pathogens [36,37]. The immune system is fundamental and plays a decisive role even before conception with a high impact on fertility [38] influencing recurrent spontaneous abortion [39,40] and promoting the genesis of a hospitable utero microenvironment for embryo implantation [41]. Table 1 summarizes gut microbiota alterations in gestational diseases.

#### 3.1.2. Vaginal Microbiota

Studies on asymptomatic reproductive-aged women had shown that the vaginal microbiota is largely dominated by lactic acid-producing bacteria, most from the *Lactobacillus* genus, suggesting a strong relationship between the acidic vaginal environment and the healthy state [42,43]. Ravel et al. analyzed, with the next-generation sequencing method, the vaginal microbiomes from 396 healthy nonpregnant women of different ethnicities. This study revealed that, in nonpregnant women, vaginal microbiota could be classified into five major types, representing the community state types (CSTs) [44,45]. Four CSTs are dominated by *Lactobacillus* spp.: *L. crispatus* in CST-I, *L. gasseri* in CST-II, *L. iners* in CST-III, and *L. jensenii* in CST-V. Instead, CST-IV is composed of facultative anaerobes as *Gardnerella*—including *Gardnerella vaginalis*—*Prevotella*, *Megasphaera*, *Sneathia*, and *Clostridiales* [46,47]. Although the prevalence of *Lactobacillus* is considered a marker of a healthy vaginal microbiome, a significant proportion of apparently healthy women have vaginal bacterial communities lacking appreciable numbers of *Lactobacillus*. It is difficult to define the composition of a universally “normal” microbiome and consequently to establish which microbial signature can predict pathological events [46]. The vaginal microbiota is subject to several transformations during a woman’s life [48] according to sexual development and sexual activity, menstruation, hygiene practice, and hormonal levels [49]. Vaginal dysbiosis could lead to a reduction in *Lactobacillus* with a shift toward a microbiome with high diversity that has been related to many pathological conditions, including acquisition and transmission [50] of sexually transmitted infections, pelvic inflammatory diseases, and adverse pregnancy events, such as PTB and premature preterm rupture of membranes (PPROM).

Pregnancy is a dynamic state characterized by several physiological events such as changes in sex hormone levels and immune system modulation. Several studies based on cultivation-independent molecular techniques demonstrated that gestation has important effects on the vaginal microbiome [51,52]. It has been observed that the microbial vaginal community shifts toward a more stable, less diverse and *Lactobacillus*-dominated state during pregnancy [53]. Freitas et al. analyzed vaginal microbial profiles in early pregnancy (11–16 weeks) in healthy women with low risk of adverse outcomes and confirmed that in these women vaginal microbiomes had lower richness and diversity, lower prevalence of *Mycoplasma* and *Ureaplasma,* and higher *Lactobacillus* abundance [49,50,51] when compared with nonpregnant women. All these modifications are probably a consequence of the increased levels of estrogen that influence vaginal epithelial maturation, producing an accumulation of glycogen that is typically used by *Lactobacillus* for lactic acid production [51]. As such, hormonal changes during pregnancy create a fertile ground for *Lactobacillus* proliferation and maintenance with increased production of high-antibacterial molecules (bacteriocins, H_2_O_2_) and lactic acid which lowers the vaginal pH [49]. Establishing the characteristics of the vaginal microbiome associated with low-risk pregnancy may help to identify which pregnancies have a higher risk of adverse reproductive outcomes, such as pregnancy loss or PTB [51], and hopefully to prevent them. Adverse pregnancy outcomes have been related to an imbalanced vaginal microbiome, as happens in bacterial vaginosis (BV), a condition characterized by a reduction in *Lactobacillus* abundance and increase in anaerobes, such as *Gardnerella vaginalis*, *Atopobium vaginae*, *Prevotella* spp., *Bacteroides* spp. These microbial changes are associated with an increase in local activation of the innate immune system and induction of the inflammatory cascade, which may induce membrane disruption with PTB or PPROM. Zheng et al. [54], who evaluated the physiological modification of vaginal microbial communities in healthy pregnant women throughout gestation, showed significant variation of *Lactobacillus* abundance among different trimesters. *L. iners* was significantly decreased in the second and the third trimesters but no significant variations in the abundance of other bacteria, such as *Gardnerella*, *Atopobium*, *Megasphaera*, *Eggerthella*, *Leptotrichia*/*Sneathia* and *Prevotella*, were detected. There are controversial data in the literature in identifying *L. iners* as beneficial or deleterious for the vaginal microbiome [54,55]. This species has unusual characteristics compared with other *Lactobacillus* spp., such as lack of growth on de Man Rogosa Sharpe agar and no production of D-lactic acid, causing a subsequent higher vaginal pH value and reduced H_2_O_2_ production [55]. Furthermore, *L. iners* is increased in women with BV and it offers less protection against BV, sexually transmitted infections and adverse pregnancy sequelae. Thus, it seems that not all *Lactobacillus* spp. can ensure a healthy vaginal state in the same entity. Several other studies have investigated other associations between adverse pregnancy outcomes and changes in the vaginal microbiome. Di Giulio et al. [52] showed an association between the *Lactobacillus*-poor vaginal CST IV, a high abundance of *Gardnerella* or *Ureaplasma* and PTB in a predominantly Caucasian cohort. A prospective study including mainly African-American women, who are known to be at higher risk of PTB [50], showed an association between premature delivery and an early significant decrease in community richness and diversity and a subsequent increased microbial instability over the gestation, thus suggesting that early vaginal microbial fluctuations could represent a marker of PTB [56]. Two studies have demonstrated an association between preterm labor and specific bacteria, such as Bacterial Vaginosis Associated Bacterium 1 (BVAB-1) in a high-risk population for PTB, established based on the history of previous PTB [57]. A longitudinal analysis of omics data from vaginal samples of a cohort of mainly African women [50] showed that preterm delivery was associated with significantly lower vaginal levels of *L. crispatus* and higher levels of BVAB-1, *Sneathia amnii,* and a group of *Prevotella* species. Furthermore, the analysis of samples collected early in pregnancy (between the 6th and 24th weeks), identified two other taxa, namely, *Megasphaera* type 1 and TM7-H1, which were significantly increased in the PTB group and, based on the analysis of vaginal fluid cytokines, were related to the overexpression of proinflammatory cytokines, consistent with the concept that microbe-induced inflammation could play a role in the induction of labor. Since these findings occur in the first few weeks of gestation, the authors suggested that this microbial and cytokine “signature” can be used as an early predictor of PTB. Instead, in normal pregnancy, the increase in stability of the *Lactobacillus* community is related to the production of antibacterial molecules (bacteriocins) and lactic acid which lowers the vaginal pH. These modifications help together to prevent ascending bacterial infections from the vagina through the cervix into the uterine cavity and finally protect the fetus from complications as PTB. However, the specific mechanisms linking the vaginal microbiome to pregnancy outcomes are still poorly understood [50,51,54]; thus, additional studies are needed with the ideal objective to identify microbial signatures associated with adverse outcomes that may eventually represent a therapeutic target.

#### 3.1.3. Endometrial Microbiota

The human uterus was traditionally considered to be sterile in absence of infections and the cervix was regarded as a perfect barrier between the vagina and the endometrial cavity. However, emerging data show the presence of bacteria in the upper reproductive tract of healthy women [58]. Mitchell et al. found that the most common genera (*Lactobacillus*, *Prevotella*) were the same in both uterus and vagina, even if the number of bacteria in the uterus was significantly lower compared to the vagina [59]. However, while the vagina is largely dominated by *Lactobacillus*, it has been observed that uterus harbors also notable percentages of *Pseudomonas*, *Acinetobacter*, *Vagococcus* and *Sphingobium*, suggesting the existence of an indigenous uterine microbiota [60]. Recent studies have investigated the role of endometrial microbiota on uterine receptivity and pregnancy outcomes, leading to results that are still controversial. Some authors have found significant differences comparing endometrial microbiota in infertile and healthy women. In particular, infertile women have a lower percentage of *Lactobacillus* [61] compared to healthy women. Moreover, Moreno et al. observed that the presence of a *Lactobacillus*-dominated microbiota (>90% of *Lactobacillus*) correlated with a higher rate of implantation in patients who underwent in vitro fertilization [62], suggesting that *Lactobacillus* may also affect blastocyst implantation. Meanwhile, other data showed no differences in implantation and pregnancy success related to the prevalence of *Lactobacillus* [63]. Due to the difficulties in obtaining samples, evidence on endometrial microbiota during pregnancy is still lacking. A pilot study by Leoni et al. analyzed uterine microbiota at a term of normal pregnancies in women subjected to caesarean delivery [64]. They found six genera in almost all patients (*Cutibacterium*, *Escherichia*, *Staphylococcus*, *Acinetobacter*, *Streptococcus*, *Corynebacterium*) which may represent a “core microbiota” of pregnancy. Interestingly, at the end of pregnancy *Lactobacillus* levels were very low (0–16%). Further studies are needed to better clarify to what extent uterine microbiota is involved both in embryo implantation and pregnancy evolution. 

#### 3.1.4. A Placental Microbiota: Real Life or Myth?

For years it has been assumed that placentas and fetuses are sterile compartments, and that microbial colonization occurs only during and after delivery. The placenta has been considered a physical and immunological barrier functioning as an interface between maternal and fetal tissues. However, this dogma has been challenged by the advent of molecular sequencing technologies that detected 16S rRNA in the placenta, amniotic fluid, and meconium [65]. Stout et al. [56], for the first time, using morphological techniques, identified the presence of nonpathogenic bacteria from placenta samples both in PTB (54%) and healthy pregnancies (26%). Aagaard et al. [66] performed a 16S ribosomal DNA-based and whole-genome shotgun (WGS) metagenomic study, characterizing a unique placental nonpathogenic microbiota composed of *Firmicutes*, *Tenericutes*, *Proteobacteria*, *Bacteroidetes,* and *Fusobacteria*, with a taxa profile similar to the human oral microbiome. Collado et al. [67] also demonstrated the presence of microbial communities in placenta and amniotic fluid of full-term pregnant women who underwent elective C-section, with a predominance of Proteobacteria (*Enterobacter*, *Escherichia*, *Shigella*, *Propionibacterium*). In this study meconium’s microbes seems to be dominated by the *Enterobacteriaceae* family, suggesting prenatally stepwise colonization. Some studies have speculated that modification in physiological placental microbiota may result in disease occurrence for both the mother and the newborn. Thus, Zheng J. et al. reported that placental microbiota significantly differs between normal-weight and macrosomic newborns, with higher abundance of *Acinetobacter*, *Bifidobacterium*, *Mycobacterium*, *Prevotellaceae*, *Dyella*, *Bacteroidales*, and *Romboutsia* in macrosomia, correlated with an elevation of IGF-1 and insulin and, conversely, with a reduction in leptin levels [68]. Furthermore, Fischer L. et al. [69] hypothesized in a recent review that the already described correlation between periodontitis and adverse pregnancy outcomes may be due to colonization and growth of oral microorganisms into the placental microbial niche. Tuominen H. et al. [70] also reported altered bacterial microbiota profile in Human Papillomavirus (HPV) positive placental samples, with an increase in *Staphylococcaceae* and a reduction in *Enterococacceae*, *Veillonellaceae*, *Corynebacteriaceae* and *Moraxellaceae* when compared with HPV-negative placentas. However, whether the changes in bacterial microbiota predispose one to or result from HPV infection was not clarified. However, the rationale of the “in utero colonization” hypothesis is based prevalently on animal models [71] and the data obtained from humans so far contrast with each other. Many researchers reported evidence refuting the existence of a placental microbiota in healthy pregnancies and its role in diseases occurrence. Starting from animal models, Malmuthuge et al. [72] analyzed the ovine fetal environment and intestine and confirmed the fetal in utero sterility during the third trimester of pregnancy. They concluded that Firmicutes and Proteobacteria reported in placental samples could be reagent contaminations. Therefore, the risk of sample contamination is the major complaint of the studies confirming the existence of a placental microbiota; thus, collecting human placentas samples in a sterile way during the delivery seems to be challenging. Accordingly, Leiby et al. [73] assert that the proofs of placental microbiome existence are not sufficient either in the setting of physiological deliveries or spontaneous PTB, due to the high probability of contamination during collection of samples. Thus, Kevin R. et al. [74] in a cross-sectional study, including placentas collected after caesarean delivery and technical controls to exclude environmental contamination, documented no differences between placenta samples and controls in term of abundance of bacterial 16S rRNA genes. Similarly, Kuperman A. et al. [75] documented no bacterial presence in 28 human placentas using the 16S rRNA gene amplification. They concluded that the placenta environment is highly probable to be sterile or else, if a placental microbiota exists, it is of extreme low biomass with an irrelevant influence on physiopathology issues. Based on current data, the evidence supporting the hypothesis of a placental microbiota is not strong enough to achieve a conclusive opinion. Further studies are necessary to support a theory rather than the other; actually, the in utero colonization hypothesis—as suggested by Perez-Muñoz et al. [76]—needs confirmation and improvement, especially regarding the bias due to sample contamination. 

### 3.2. Major Adverse Pregnancy Outcomes and the Role of Dysbiosis

#### 3.2.1. Gestational Diabetes Mellitus

The rationale behind maternal metabolic adaptations is to ensure continuous energy supply and nutrients to the developing fetus even during mother fasting periods. In the early stage of pregnancy, there is a reduction in insulin sensitivity in peripheral body districts (mainly muscles and adipose tissue) despite a normal insulin secretion. This condition is called insulin resistance and helps to increase substrates availability for the fetus and its nutritional needs. However, on the other hand, peripheral tissues show a reduced response to insulin with the consequent increase in maternal postprandial glucose values. In women whose pancreatic function is insufficient to overcome insulin resistance, this adaptation could lead to GDM. GDM is a condition characterized by altered glycemic values which occur during pregnancy in women who were not diabetic before. Risk factors for GDM include overweight, previously GDM, a family history of type 2 diabetes and polycystic ovary syndrome [77]. GDM has always raised concerns due to the related adverse events [78] as large-for-gestational-age newborns and macrosomia [79], leading to further birth complications [80] such as caesarean delivery, shoulder dystocia (with brachial plexus injury and fracture) [81], PTB, hyperbilirubinemia, and pre-eclampsia. Microbiota could play a major role in the pathogenesis of GDM. As previously described, the microbiota can follow maternal body transformations, including metabolic ones. Several studies have evaluated how some of the metabolic changes underlying GDM are often accompanied by such changes in microbiota [82] in several niches where specific microbial alterations could be used as a disease biomarkers. Zheng et al. [83] analyzed how dynamic changes of gut microbiota, from the first trimester (T1) to the second trimester (T2), could be correlated with later development of GDM. Particularly, women who developed GDM exhibited fewer taxonomic and functional shifts in gut microbiota from T1 to T2 compared with normoglycemic women. Usually, *Blautia*, *Rothia*, and *Bilophila* are positively associated with inflammation, insulin resistance, and impaired glucose tolerance while *Bacteroides*, *Parabacteroides,* and *Acinetobacter* are negatively associated with these alterations. Interestingly, in this study there were no differences in the abundances of these microbes from T1 to T2 in GDM women, suggesting that these gut microbiota may contribute to enhanced insulin resistance in early pregnancy in this group [83]. The oral *Neisseria*/*Leptotrichia* ratio positively correlates to fasting blood glucose values that reflect the daily secretory capacity of basal insulin [84]; a low intestinal *Faecalibacterium*/*Fusobacterium* ratio corresponds to high blood glucose values [85] and a high vaginal *Prevotella*/*Aerococcus* ratio correlates to high blood glucose values [85]; GDM mothers showed a positive correlation between maternal fasting glucose and *Acinetobacter* abundance and a negative correlation with *Prevotella* [85]. Guangyong et al. [86] investigated 52 pregnant women for differences in gut microbiota between GDM patients with successful glycemic control (GDM1) and patients who failed to achieve glycemic control (GDM2) with lifestyle modifications. They showed that *Blautia* and *Eubacterium* were enriched in GDM2, whereas *Faecalibacterium*, *Subdoligranulum*, *Phascolarctobacterium*, and *Roseburia* were enriched in the GDM1 group, underlining how gut microbiota could participate in the definition of a successful glycemic control, through the peroxisome proliferator-activated receptor (PPAR) signaling pathway. However, in the literature, studies are still controversial. Ferrocino et al. [87] showed that from the second to the third trimesters of pregnancy, there is a higher α-diversity in GDM microbiota with an increase in Firmicutes and Bacteroidetes and reduction in Actinobacteria compared with non-GDM women; other authors have found a positive correlation between *Prevotella* increase and HbA1c levels [88]. Finally, some data suggested that the modifications of maternal microbiota can be transmitted in utero to the fetus through the vertical line [89], causing the predisposition of the newborn to develop metabolic syndrome during childhood [90,91]. 

#### 3.2.2. Pre-eclampsia

PE is a pregnancy or postpartum pathology characterized by the new onset of hypertension (systolic blood pressure >140 mmHg and/or diastolic blood pressure >90 mmHg) with or without proteinuria and multiorgan dysfunction [92]. At the basis of the pathogenesis of PE, there is an altered flow exchange between the uterus and placenta with a consequent reduction in oxygen supply to placental and fetal tissues [93]. The low oxygen exchanges determine oxidative stress and placental ischemia. Local placental alterations stimulate the release of antiangiogenic factors in the systemic maternal blood circulation with successive vascular dysfunction (arterial hypertension and proteinuria) [94]. However, some aspects of the genesis of PE remain unclear. Recent studies have investigated the role of gut microbiota in the onset of this condition. Women with PE showed high levels of opportunistic pathogens (i.e., *Fusobacterium* and *Veillonella*) and less beneficial bacteria (i.e., *Faecalibacterium* and *Akkermansia*) compared to control [95]. This microbiota imbalance is also related to blood pressure levels and markers of kidney dysfunction. Liu et al. described significant differences in the composition of gut microbiota of pregnant women with and without PE, suggesting a possible relationship between dysbiosis and the development of the disease. Lv et al. [96] analyzed gut microbiota changes before and after delivery in early-onset PE and normotensive women, showing a correlation with both maternal blood pressure levels and newborn features (e.g., Apgar score or newborn birth weight). In fact, they found an association between high maternal blood pressure and liver enzyme levels with increased levels of *Blautia* and *Ruminococcus* (also associated with obesity and type 2 diabetes) [97,98] and *Bilophila* and *Fusobacterium*. On the other hand, they showed that in PE women there was a reduction in *Faecalibacterium*, *Methanobrevibacter* and *Akkermansia* compared to controls. Reduced levels of short-chain fatty acid (SCFA)-producing bacteria (*Faecalibacterium*) and anti-inflammatory bacteria (*Akkermansia*) would contribute to increase the risk of high blood pressure levels [99]. In fact, SCFAs are known to reduce systemic blood pressure [100] and inflammation both in mothers and newborns [101]. Chronic inflammation plays an important role in the pathogenesis of PE and microbiota-derived metabolites, such as SCFAs and lipopolysaccharides (LPSs), can contribute to the balance between pro- and anti-inflammatory statuses. In particular, SCFAs are known to reduce both systemic blood pressure and inflammation. LPS, instead, is a well-known inflammatory factor and it has been observed that in PE patients fecal and plasma concentrations of LPSs [102] are higher than in healthy controls. Indeed, Wang et al. [103] found that in PE women in the third trimester there is an increase in Bacteroidetes, Gram-negative bacteria which contribute to LPS biosynthesis, and a significant reduction in Firmicutes, Gram-positive bacteria able to produce SCFAs. Moreover, microbiota imbalances in PE women are related not only with blood pressure levels but also with markers of kidney dysfunction [95]. PE is not only a pregnancy disease; women that have an episode of PE show high predispositions to cardiovascular and renal diseases for their entire lives. For these reasons, it appears of key importance to understand the role of microbiota and other factors involved in the etiopathogenesis of PE. 

#### 3.2.3. Preterm Birth

The major scientific evidence on the correlation between dysbiosis and negative pregnancy outcomes are focused on PTB. PTB refers to a delivery that occurs before 37 weeks of gestation. There are several risk factors/predictors for this condition even if the exact etiology is not completely understood yet [104]. It is known that up to 70–80% of PTBs are spontaneous and the remaining 20 to 30% are iatrogenic. It is possible to identify several risk factors that predispose one to PTB, such as cigarette smoking, unbalanced diet [105], excessive weight gain/loss [106], previous episodes of PTB [105], short cervical length [107,108] detected in the second trimester, decidual hemorrhage, and maternal or fetal stress [109]. There is a consensus about the protective role played by *Lactobacillus* spp. in the vaginal microbiota of healthy reproductive-age women. An alteration of this vaginal microbiota homeostasis during pregnancy seems to be correlated with PTB. Shi et al. [110], using a 16s ribosomal RNA sequence method, analyzed samples of vaginal microbiota from 64 pregnant women to evaluate a possible correlation between premature labor, preterm delivery, and vaginal microbiota alterations. They found no differences in vaginal biomarkers and in the prevalence of *Lactobacillus* and other species during pregnancy between threatened premature labor and nonthreatened premature labor groups. However, the loss of *Lactobacillus* spp. in vaginal microbiota during pregnancy could predict preterm delivery. Kacerovsky et al. [111] showed the association between *L. crispatus*-dominated cervical microbiota and a lower risk of intra-amniotic complications and early-onset sepsis of newborns with PPROM in 311 women. The presence of *L. crispatus* in the cervical microbiota seems to protect pregnant women from intra-amniotic infections. They also showed that *Ureaplasma* spp. in vaginal microbiota could be a risk factor for preterm delivery. In fact, *Ureaplasma* spp. represent the most commonly identified bacteria in the amniotic fluid in PPROM. In a large cohort study, Doyle et al. [112] evaluated the correlation between the placental and fetal tissue microbiome and birth outcomes. They showed that specific combinations of bacteria were associated with severe chorioamnionitis (*Mycoplasmataceae*, *Leptotrichiaceae,* and *Veillonaceae*), shorter duration of pregnancy (*Fusobacterium nucleatum*, *Ureaplasma* spp. and *Gemella asaccharolytica*) and smaller newborn size (*Sneathia sanguinegens*, *Prevotella copri*, *Lachnospiraceae* spp., and *Phascolarctobacterium succinatutens*). Kindinger et al. [113] focused on the importance of the species-specific benefits of different *Lactobacillus* spp. during pregnancy to predict the risk of preterm/term birth. They showed how *L. crispatus* abundance in maternal vaginal microbiota seems to reduce the risk of PTB compared to *L. iners,* which is linked with an increased risk of preterm delivery (<34 weeks). One of the most validated etiologies in the pathogenesis of PTB is certainly the infection via the ascending pathway from the vagina. Bacterial vaginosis and vaginitis, indeed, represent one of the major known risk factors for PTB. From the vagina, bacteria could reach the fetal environment and determine amniotic infections that consequently activate the inflammatory cascade [114]. A novel hypothesis proposed by Lokken et al. [115] focused on the scant efficacy of the treatment of vaginal infections during pregnancy on prevention of spontaneous PTB. They suggested that spontaneous PTB could be mostly related to vaginal microbiota at the time of conception, more than they are to vaginal infections in later phases. Dysbiosis in such an early phase could compromise the protective effects of cervical mucus, leading to microbial colonization of the endometrial surface before fetal membrane development, causing low-level inflammation in the decidua, placenta, and fetal membranes which finally could determine a chronic inflammatory status that it is associated with PTB and also with low birth weight, sepsis, bronchopulmonary dysplasia, and neonatal mortality. The production of active cytokines and enzymes stimulates the maternal inflammatory response with the premature activation of pathways involved in labor. The premature or pathological activation of NF-kB stimulates myometrial contractions, cervical remodeling, membrane rupture and consequent PTB [116]. Prince et al. [117] hypothesized that placental membranes would retain a microbiome community that varies in association with PTB and chorioamnionitis. You et al. [118] showed that the number and composition of bacteria in blood samples of women with PTB is different from women with term delivery. Particularly, several taxa, such as *Bacteroides*, *Lactobacillus*, *Delftia*, and *Pseudomonas,* exhibited differential enrichments between women with and without PTB. In fact, in PTB there is an increased number of Firmicutes and Bacteroidetes and a decreased number of Proteobacteria compared to controls. The ascension of pathogens from the vagina does not represent the only mechanism at the basis of PTB pathogenesis. Hematogenous spread of microbes with consequent placental colonization has also been proposed as a potential secondary route of invasion and infection leading to PTB [119]. The similarity between the oral and placental microbiome suggests that the placental microbiome becomes colonized primarily as the result of hematogenous bacterial spread via the circulation [120]. However, contradictory data on maternal periodontitis, that could represent an independent risk factor for PTB, is present in the literature. Hongyu et al. [121] hypothesized that periodontitis could act as a distant reservoir of microbes and inflammatory mediators contributing to PTB induction through secretion of proinflammatory cytokines [122,123,124,125]. In conclusion, dysbiosis seems to be related to PTB; however, further studies are necessary to better understand the correlation between this pregnancy complication and the specific microbiota alteration. 

## 4. Discussion and Future Outlooks

Recent studies have shown the presence of microbial material in utero, endometrium, placenta, amniotic fluid, and meconium, questioning the “sterile womb paradigm” and fortifying the “in utero colonization hypothesis”. Results are still contrasting in the literature, but the eventual confirmation of the presence of microbiota within the maternal–fetal interface could open new perspectives strategies in the treatment and prevention of pregnancy pathologies and complications. Microbiota can regulate our immune, endocrine, and metabolic systems during our entire life so it is not surprising that it may also govern some of the pathogenetic mechanisms of pregnancy-related pathologies and complications. However, further investigations are necessary to overcome some of the bias that is currently present in the literature.

In the future, a better understanding of the microbial signature of healthy and pathological pregnancies will help to identify women at risk of pregnancy-related complications in an early stage of pregnancy and, maybe, also before conception, using non-invasive methods (i.e., fecal or salivary microbial characterization). This improved early diagnosis will also offer new preventive strategies based on microbiota modulation through a personalized nutritional plan including the use of prebiotics and probiotics, selected on the specific individual dysbiosis, with the possibility of monitoring the effects on microbiota during the treatment. Based on the vertical transmission of microbiota, the modulation of maternal microbiota could also help to reduce the risk of the newborn to develop noncommunicable diseases, such as metabolic diseases, later in life and could be considered the first form of antenatal primary prevention. Future research will evaluate these aspects and explore the real potential of microbiota modulation.

## Figures and Tables

**Table 1:** Alterations of maternal gut microbiota in gestational diseases.

	Gestational Diabetes	Pre-Eclampsia	Pre Term Birth	Birth Complications
Increased	Genus *Blautia* *Rothia* *Bilophila* *Eubacterium * *Phascolarctobacterium Fusobacterium* Species *Roseburia Subdoligranulum*	Phylum Bacteroidetes Genus *Fusobacterium* *Veillonella* *Blautia* *Ruminococcus* *Bilophila*	Genus *Ureaplasma * Species *Fusobacterium nucleatum * *Gemella asaccharolytica*	Genus *Mycoplasmataceae ^* *Leptotrichiaceae ^* *Veillonaceae ^ * Species *Sneathia sanguinegens #* *Prevotella copri #* *Lachnospiraceae* spp # *Phascolarctobacterium succinatutens #*
Reduced	Genus *Bacteroides* Parabacteroides Acinetobacter * *Fecalibacterium* *Prevotella*	Phylum Firmicutes Genus *Faecalibacterium* *Akkermansia* *Methanobrevibacter*		

* Controversial data in literature, see text for details. ^ Associated with increased risk of choriamnionitis. # Associated with increased risk of small newborn size.

## Data Availability

Not applicable.
